# Investigation of sorptive interactions between volatile organic compounds and supramolecules at dynamic oscillation using bulk acoustic wave resonator virtual sensor arrays

**DOI:** 10.1038/s41378-024-00729-x

**Published:** 2024-07-17

**Authors:** Zilun Wang, Zeyu Zhao, Suhan Jin, Feilong Bian, Ye Chang, Xuexin Duan, Xiangdong Men, Rui You

**Affiliations:** 1 State Key Laboratory of NBC Protection for Civilian, Beijing, 102205 China; 2 State Key Laboratory of Precision Measuring Technology & Instruments, Tianjin University, Tianjin, 300072 China; 3 School of Instrument Science and Opto-Electronics Engineering, Beijing Information Science and Technology University, Beijing, 100192 China

**Keywords:** Chemistry, Electrical and electronic engineering

## Abstract

Supramolecules are considered as promising materials for volatile organic compounds (VOCs) sensing applications. The proper understanding of the sorption process taking place in host-guest interactions is critical in improving the pattern recognition of supramolecules-based sensing arrays. Here, we report a novel approach to investigate the dynamic host-guest recognition process by employing a bulk acoustic wave (BAW) resonator capable of producing multiple oscillation amplitudes and simultaneously recording multiple responses to VOCs. Self-assembled monolayers (SAMs) of β-cyclodextrin (β-CD) were modified on four BAW sensors to demonstrate the gas-surface interactions regarding oscillation amplitude and SAM length. Based on the method, a virtual sensor array (VSA) type electronic nose (e-nose) can be realized by pattern recognition of multiple responses at different oscillation amplitudes of a single sensor. VOCs analysis was realized respectively by using principal component analysis (PCA) for individual VOC identification and linear discriminant analysis (LDA) for VOCs mixtures classification.

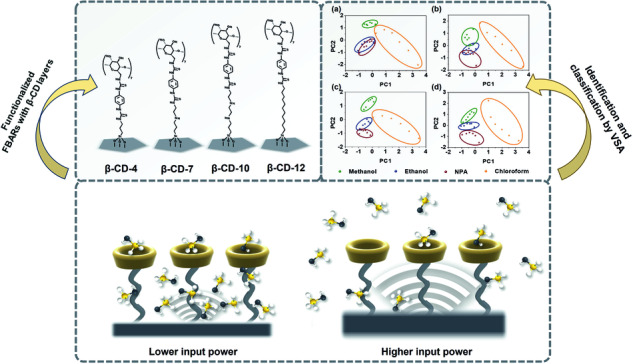

## Introduction

Volatile organic compounds (VOCs) are carbon based chemicals classified on the basis of boiling point, consisting of different types of components with various structural and physical characteristics^1,2^. Over the past decade, detection of VOCs has been a great challenge in environmental monitoring^3,4^, food evaluation^5–7^, industrial safety^8^ and disease diagnosis^9,10^. Extensive research has shown that the main difficulty of analyzing VOCs lies in identifying a specific component from complex samples and classifying the mixtures^11,12^.

At present, one effective solution to such kind of problems is the electronic nose (e-nose) system^13,14^. A conventional e-nose usually comprises a multisensor array functionalized with vapor recognition materials, and provides pattern recognition processing for VOCs discrimination^15,16^. With recent developments in micro/nanoelectromechanical systems (MEMS/NEMS) device fabrication, there has been an increasing interest in the use of micro/nano devices as chemical sensors in the sensing array. For each chemical sensor, suitable surface functionalization is required to improve its crucial parameters (e.g., sensitivity, selectivity, and response time) for a better recognition capacity^17^. Thus, of particular concern are two aspects: 1) the sorptive interactions between vapor molecules and recognition materials and 2) the association between recognition materials and the working mechanism of the sensor.

Supramolecules are self-associated molecules mainly combined by weak noncovalent interactions^18,19^. Due to the flexibility and sensitivity for various chemical species, supramolecular coatings have become promising materials for sensing applications, including DNA^20^, proteins^21,22^ and metal ions^23,24^. In recent years, these materials have also been widely used in VOCs chemical sensors for selectivity improvement owing to the specific interaction between “hosts and guests”^25–27^. A series of studies are mainly concerned with size and structure effect on interfacial molecular recognition, e.g., cavity size^28^, central metal atom^29^ and functional groups^30,31^. In these cases, quartz crystal microbalance (QCM) has been proved to be a suitable sensing component since an acoustic wave device is capable of comparing the sensitivity or selectivity of different supramolecular coatings by recording the mass of adsorbed analytes. While these studies have effectively contributed to the selection of recognition materials, however, there is still a necessity to properly understand the dynamic sorption process taking place in the host-guest recognition^32^.

Generally, the adsorbed mass on an acoustic wave sensor induces its oscillation frequency variation, and the oscillation amplitude is usually kept constant^33^. In this work, a novel strategy is proposed to investigate the host-guest recognition process at dynamic oscillation amplitude using film bulk acoustic resonators (FBARs) modified by a self-assembled monolayer (SAM) of β-cyclodextrin (β-CD). FBAR is a type of bulk acoustic wave (BAW) devices, which can be mass fabricated owing to their miniaturization and CMOS-compatible manufacture^34^. According to the Sauerbrey equation^35^, high operation frequency (usually at GHz) gives FBAR a much higher sensitivity than conventional acoustic wave sensors^36^. A series of results for vapor sensing have been reported using FBAR sensors^37–40^ and it has been demonstrated that FBAR is able to provide multiple sensor outputs, which facilitates the selective detection and identification of VOCs^27,41^. In particular, FBAR is mainly operated in a thickness extensional mode. The generated longitudinal acoustic wave, which propagates into the gas-surface interface, can be rationally designed for the dynamic sorption process.

In this work, a three-dimensional finite element method (3D FEM) analysis was first applied to investigate the positive correlation between the oscillation amplitude and input power of FBAR. Then, four β-CD SAMs with different chain lengths were respectively assembled on four FBAR sensors to further investigate the gas-surface interactions. The sensors were independently exposed to VOC analytes in the manner of recording frequency responses at a dynamic input power, thus the gas-surface interactions can be analyzed with respect to oscillation amplitude and SAM length. Furthermore, it can be found that each individual FBAR sensor is capable of producing multiple responses to analytes at multiple oscillation amplitudes, forming a novel virtual sensor array (VSA) type e-nose, which uses a single sensor to simulate the response spectrum of a sensing array^42,43^. Finally, an identification of four VOCs and a classification of vapor mixtures was investigated based on the VSA.

## Material and methods

### Chemicals

VOCs (methanol, ethanol, NPA and chloroform) utilized in this work were purchased from Real&Lead Chemical Corporation (Tianjin, China) and the purity all reached HPLC. (3-aminopropyl) triethoxysilane (APTES) and p-phenylene diisothiocyanate (PDC) were purchased from Aladdin Industrial Corporation (Shanghai, China). N-[3-(trimethoxysilyl) propyl] ethylenediamine (TMESPE) and 3-[2-(2-aminoethylamino) ethylamino] propyl-trimethoxysilane (AEAPTES) were purchased from HEOWNS Biochemical Technology (Tianjin, China). 11-(triethoxysilyl)-1-undecanamine (AUTES) was purchased from Alfa Aesar (Ward Hill, MA, USA). All chemicals were used as received without further purification. Per-6-amino-β-cyclodextrin (per-6-ABCD) was synthesized according to previous results^44^.

### Device fabrication and functionalization

**Fig. 1:**
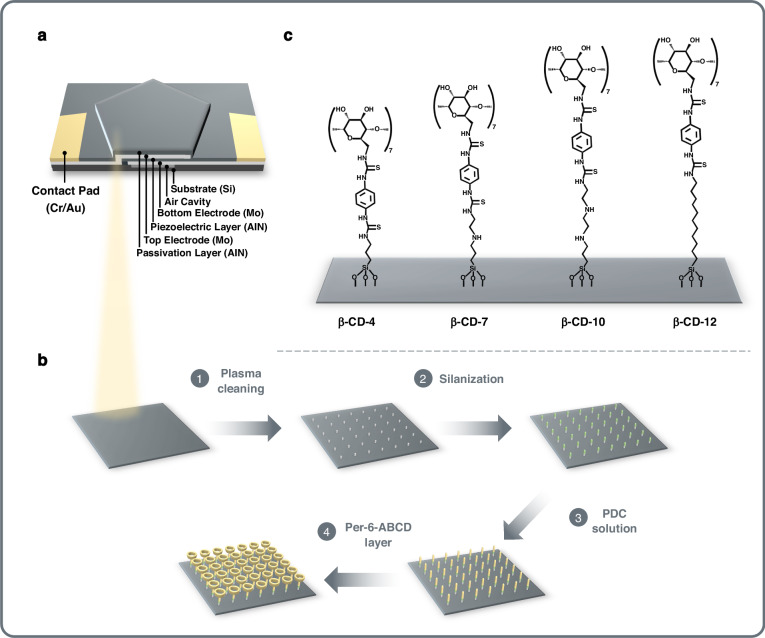
Design Architecture of the Sensor Element. Schematic illustration of (**a**) a FBAR sensor, (**b**) functionalization of FBARs with β-CD layers and (**c**) four β-CD SAMs used in this work

2.44 GHz FBARs (Fig. 1a) were conveniently fabricated with a standard MEMS fabrication process as described previously (shown in Figure S1)^38,41^, First, an air cavity was etched on a silicon wafer by deep reactive ion etching. Subsequently, the cavity was filled with phosphosilicate glass (PSG) as a sacrificial layer via chemical vapor deposition. A sandwiched structure of Mo/AlN/Mo was then deposited as the bottom electrode, piezoelectric layer, and top electrode, respectively. After the deposition of a passivation layer (AlN), a Cr/Au composite film was then deposited and patterned to form contact pads. Finally, PSG in the cavity was removed by immersing the chip in a diluted HF solution.

Figure 1b shows the process scheme of the functionalization of FBARs with β-CD. After fabrication, FBARs were oxidized in exposure to air plasma for 5 min by a plasma cleaner (YZD08-2C, SAOT, China). Silanization with APTES, TMESPE and AEAPTES was achieved by vapor phase deposition of silylating reagents under reduced pressure in a heated chamber (YES-LabKote, Yield Engineering Systems, USA). Silanization with AUTES was achieved by immersing oxidized FBARs in freshly prepared AUTES solution (1 mM in ethanol) for 6 h as described by Giraud et al.^45^. Subsequently, amino-silanized FBARs were exposed to a PDC solution (1 mM in ethanol) at 40 °C for 1 h, followed by being rinsed with ethanol and dried in a nitrogen stream. The β-CD layer was obtained by immersion of FBARs in a 5 mM aqueous solution of per-6-ABCD at 40 °C for 1 h, followed by washing with deionized water and drying in a nitrogen stream.

### Surface characterization

The β-CD functionalizations coating on AlN substrates were characterized by Fourier transform infrared (FT-IR) spectrometer (Vertex 70 v, Bruker Optics, Ettlingen, Germany) and atomic force microscopy (AFM, Dimension Icon, Bruker, Rheinstetten, Germany) in tapping mode.

### VOCs detection system

The homemade VOCs detection setup has been described previously (shown in Figure S2)^46^. In brief, it consists of two parts, a dual-line vapor delivery system and a testing system. In the delivery system, vapors were bubbled out of the liquid with carrier nitrogen (99.999%). VOCs in varied concentrations were prepared by adjusting the flow rate of nitrogen from the dilution line via a mass flow controller (MFC, 5850e, Brooks, USA). Experiments were performed with exposure to vapors at concentrations in terms of P/P_0_ from 0.1 to 0.6, where P and P_0_ represent the partial pressure and the saturated vapor pressure of the vapor of interest. In the testing system, FBAR sensors were wire-bonded onto an evaluation board, packaged in a stainless-steel chamber, and connected to a vector network analyzer (VNA, E5071C, Agilent, USA) for frequency measurements. Different input powers (10 dBm, 5 dBm, 0 dBm, and -5 dBm) were applied by the VNA. A MATLAB program was developed to alternatively record the real-time frequency variation of a FBAR at different input powers in an eight-second cycle (2 s for each input power).

## Results and discussions

### Oscillation amplitude of FBAR effected by input power

**Fig. 2:**
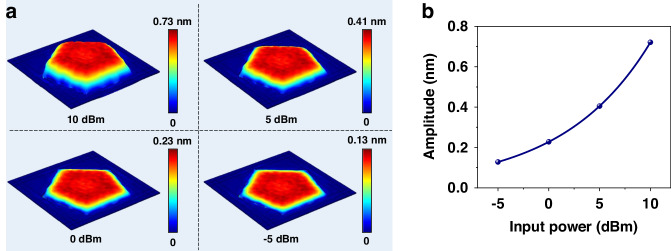
3D Finite Element Method (FEM) Simulation Outcomes for FBAR Oscillations. 3D FEM simulation results of FBAR’s oscillations: (**a**) distribution of oscillation amplitude induced by input powers of 10 dBm, 5 dBm, 0 dBm and −5 dBm, (**b**) maximum oscillation amplitudes versus different input powers

A typical gravimetric FBAR comprises a sandwiched structure of a piezoelectric layer interposed between two electrodes to generate acoustic waves (Fig. 1a). The acoustic energy distribution of an FBAR device is correlated positively with the input power, and such effect has been applied in the hypersonic poration of cells, vesicles and supported lipid bilayers^47,48^. To further investigate the effect, a 3D FEM analysis was applied to compare the resonant oscillations of FBAR at different input powers. Figure 2a shows the simulation result of a 2.44 GHz FBAR’s oscillation with input powers of 10 dBm, 5 dBm, 0 dBm and −5 dBm, and the simulated color patterns indicate the distribution of oscillation amplitude at resonance. Obviously, the amplitude is larger in the central region of the resonator, which is due to the boundary condition of zero displacement as previously described^49,50^. Figure 2b summarizes the maximum oscillation amplitudes of FBAR’s resonance at four input powers. The result shows that the higher values of input power imply the higher values of oscillation amplitude, which is consistent with an exponential relationship.

It is worth noting that the oscillation amplitude is at subnanometer level. Such molecular scale of oscillation is beneficial for investigating the sorptive interactions between VOC molecules and supramolecules. Therefore, we used FBAR chips functionalized with supramolecules for the VOCs sorption testing. Figure 1c shows the four cyclodextrin SAMs based on amino-silane reagents with different chain lengths of 4, 7, 10, and 12. Accordingly, the supramolecules are labeled as β-CD-4, β-CD-7, β-CD-10, and β-CD-12 hereafter. It has been reported that such stable cyclodextrins allow long-term storage of the functionalized sensors^51^. To realize a dynamic operation of multiple oscillation amplitudes of FBAR sensors, we applied different input powers switched by the VNA.

### Characterization of supramolecules

**Fig. 3:**
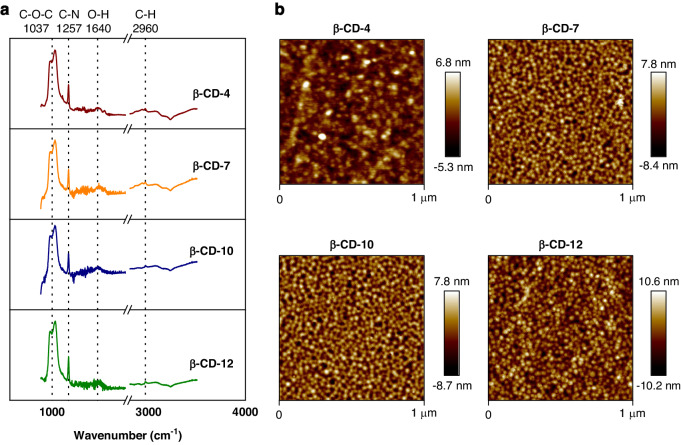
Characterization of Surface-Sensitive Materials. (**a**) FT-IR spectroscopy results and (**b**) AFM images of four β-CD SAMs

FT-IR and AFM were used to characterize the β-CDs on AlN substrates. FT-IR spectrums of four supramolecules were quite similar (Fig. 3a). The peaks at about 1037, 1257, 1640, and 2960 cm^−1^ can be indexed to C-O-C, C-N, O-H and C-H groups respectively^52^, which are consistent with the molecular structures of β-CD SAMs. The surface morphology of β-CDs were further investigated by AFM (Fig. 3b). Average heights of four SAMs are estimated to be 3.05 ± 0.52 nm, 6.90 ± 0.76 nm, 9.43 ± 1.14 nm, and 10.71 ± 1.31 nm, which corresponds with their chain lengths and indicates the uniform coatings on FBAR sensors.

### Sorption process of VOCs on β-CDs with different oscillation amplitudes

**Fig. 4:**
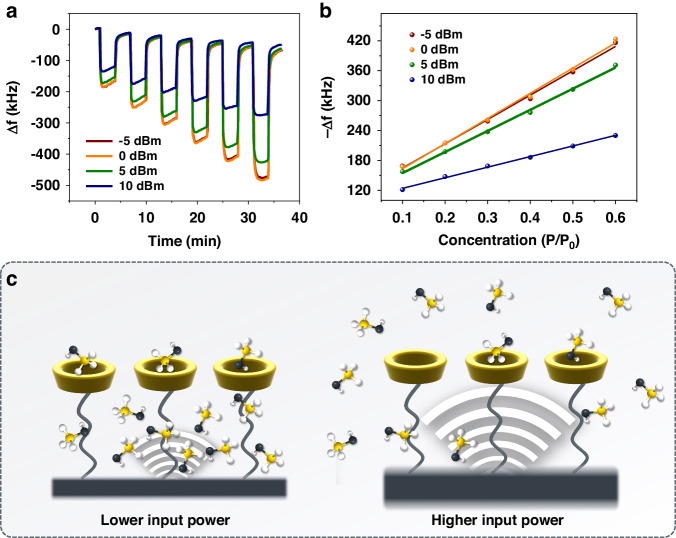
Gas Detection Efficacy at Varied Input Power Levels. **a** Plot of real-time responses of the β-CD-4 functionalized sensor to chloroform at different concentrations from P/P_0_ = 0.1 to 0.6. **b** Linear fits of responses of the sensor to chloroform. **c** Schematic of sorption process of vapor molecules at lower and higher input powers

To investigate the gas-surface interactions under different oscillating conditions, we applied the β-CD functionalized FBAR sensors for the sensing of four VOCs (methanol, ethanol, NPA, and chloroform). Figure 4a shows responses of the β-CD-4 functionalized sensor to chloroform vapor from P/P_0_ = 0.1 to 0.6 at four different input powers (10 dBm, 5 dBm, 0 dBm and −5 dBm). The linear relationship between concentration and frequency change within this concentration range (Fig. 4b) indicates an unsaturated adsorption of VOCs in the SAM layer. Besides, it can be observed that the sensor has weaker responses to VOCs at high input power. We assume that the higher oscillation of the resonator facilitates the desorption of vapor molecules from β-CD-4 layer, and thus, the response is weaker (Fig. 4c). While at lower input powers, such as 0 dBm and −5 dBm, the oscillation effect is negligible due to the relatively close amplitudes (Fig. 2b), which makes responses at such input powers quite similar.

**Fig. 5:**
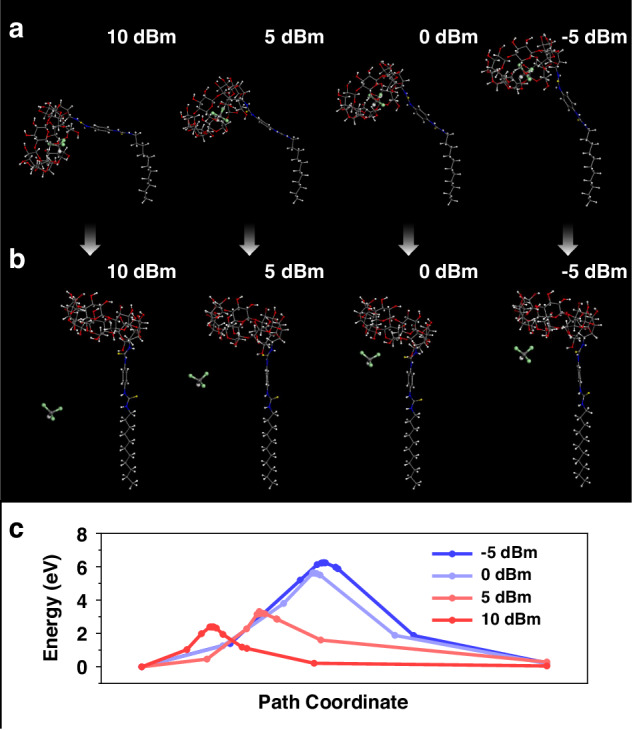
Transition State Exploration for Diverse Oscillation Amplitudes. The (**a**) initial state, (**b**) final state and (**c**) minimal energy paths of the transition state search calculations for the sorption process of VOCs with different oscillation amplitudes

For the purpose of further validating the supposition, a transition state search was performed between an initial state where a chloroform molecule adsorbed on the cavity of a bent β-CD-12 (Fig. 5a) and a final state where the molecule desorbed from an unbent β-CD-12 (Fig. 5b). The bent and unbent β-CD SAMs were used to simulate extreme molecular geometries under dynamic oscillation conditions. And four bending amplitudes correspond to the oscillation amplitudes described in Fig. 2 were used to simulate molecular geometries under oscillations with input powers of 10 dBm, 5 dBm, 0 dBm and −5 dBm, respectively. Each transition state, including the adsorption site was refined through geometry optimization. Figure 5c depicts the calculated minimal energy paths of the transition state search. The calculated energy barriers of chloroform molecules desorption from β-CD SAMs are negatively correlated with their assumed oscillation amplitudes. It can therefore be concluded that vapor molecules prefer to desorb from β-CD SAMs at higher input powers, which further proves the supposition in Fig. 4c.

**Fig. 6:**
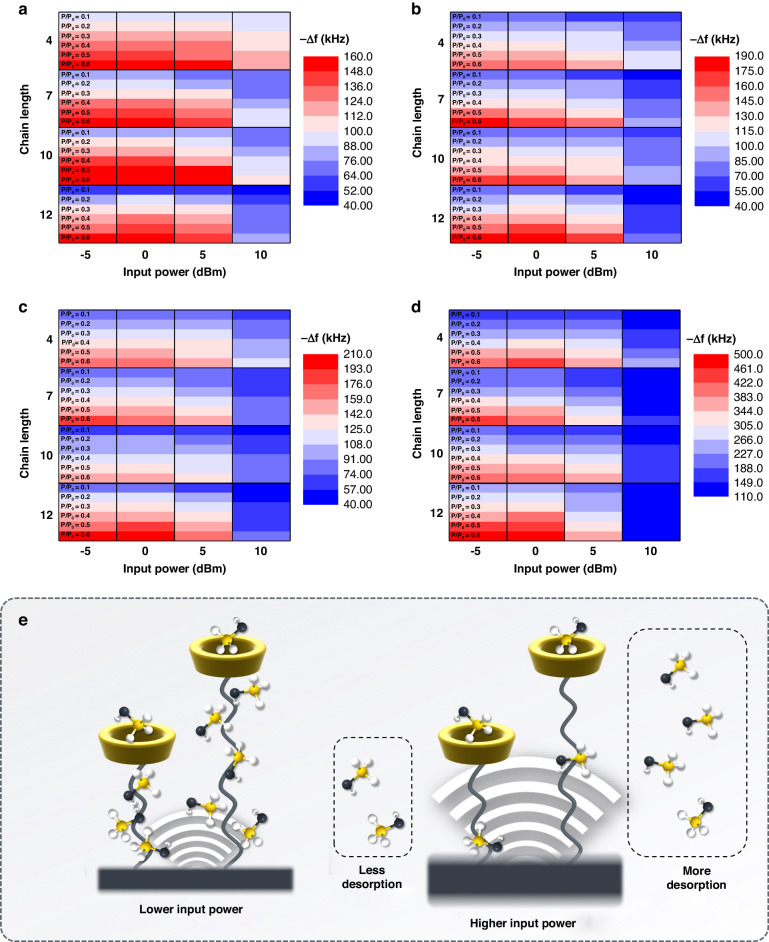
Frequency Response Heat Maps for Sensors with Varied Chain Lengths Exposed to Different VOCs. Heat-maps of frequency responses of the SAM-functionalized sensors upon exposure to (**a**) methanol (**b**) ethanol (**c**) NPA and (**d**) chloroform at concentrations P/P_0_ = 0.1 to 0.6. **e** Schematic of sorption process of vapor molecules on long chain and short chain SAMs functionalized sensors at lower and higher input powers

Similar trends were observed for all FBAR sensors. Heat-maps in Fig. 6 summarize responses of sensors with different chain lengths of supramolecules to four VOCs at concentrations from P/P_0_ = 0.1 to 0.6. The oscillation effect due to higher input power facilitates the desorption of VOCs molecules from all β-CD SAMs. Compared with alcohol vapors, chloroform gets higher responses, which is probably attributed to high molecular weight. Similarly, in alcohol vapors, the proportional relation of responses with carbon chain lengths can also be related to molecular weight.

It is worthwhile to point out that, SAMs with longer chain length tend to facilitate the differentiation of responses at different input powers. For instance, in Fig. 6d, sensor with longer SAM functionalization gets stronger responses to chloroform at low input powers (0 dBm and −5 dBm) and weaker responses at high input power (10 dBm), while sensor with short SAM functionalization behaves inversely. The trend was also observed for other three vapors. It is assumed that a longer carbon chain provides more adsorption sites for VOC molecules, which makes longer SAM-functionalized sensors get stronger responses at low input powers. However, the affinity constant of outside-cavity adsorption is often lower than that of inside-cavity adsorption as described before^27^. Therefore, molecules tend to desorb from outside of the β-CD cavity rather than from inside of the cavity with the oscillation of resonator, which makes longer SAM-functionalized sensor get weaker responses at higher input powers (Fig. 6e).

Furthermore, the manner of recording frequency responses at different input powers forms a novel VSA type e-nose, in which an individual FBAR can produce multiple responses based on the dynamic operation to simulate the response spectrum of FBAR arrays. Conventional e-nose systems using sensing arrays tend to suffer from problems such as additional power supplies, difficult modification processes^53^ and extra breakdown possibilities^43^, which make such devices far from ideal for tight cost, simple manufacture as well as long-term usage. The VSA sensor similarly satisfies the demand for VOCs analysis and ideally optimize the characteristic of a sensing system. In particular, the enhancement of response differentiations potentially facilitates the discrimination capacity of the longer SAM-functionalized FBAR as a VSA sensor.

### VOCs identification results of VSA sensors

**Fig. 7:**
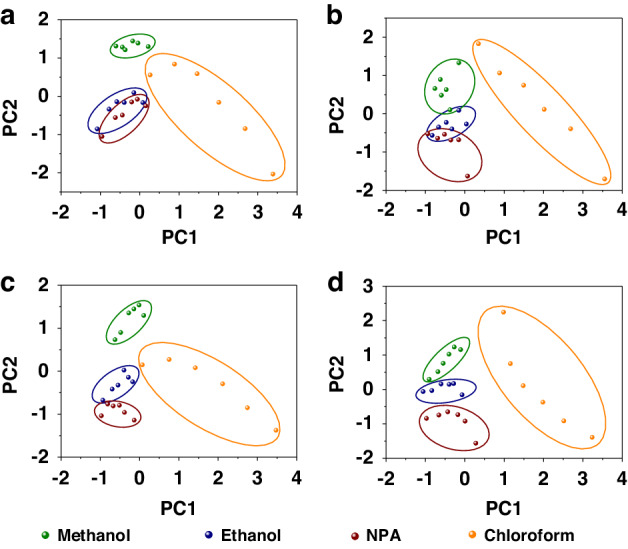
Principal Component Analysis (PCA) of VOCs Utilizing VSA Sensors. PCA identification results of VOCs based on VSA sensors functionalized with (**a**) β-CD-4, (**b**) β-CD-7, (**c**) β-CD-10, and (**d**) β-CD-12

As assumed above, the differentiation of responses at different input powers gives an insight into the possibility of VOCs analysis by this type of VSA sensor. In order to demonstrate the identification capacity of four VSA sensors, we performed principal component analysis (PCA)^54^, a discrimination method of multivariate statistical analysis, on the datasets of normalized frequency shifts (Δf) displayed above. Figure 7 shows PCA discriminations of four VOCs based on the sensors. For all the sensors, it is easy to identify clear clusters between alcohol vapors and chloroform. However, the three alcohol vapors with similar properties are hardly distinct from each other for shorter SAMs (β-CD-4 and β-CD-7) functionalized sensors. Correspondingly, the best separation is achieved with the β-CD-12 functionalized sensor. The results indicate that, longer SAM-functionalized VSA sensors have stronger discrimination capacity of VOCs due to their enhancement of response differentiations. The discrimination capacity also provides the input power-based VSA sensor potential to become an integrated VOCs sensing system.

### Mixture Classification with the VSA sensor

**Fig. 8:**
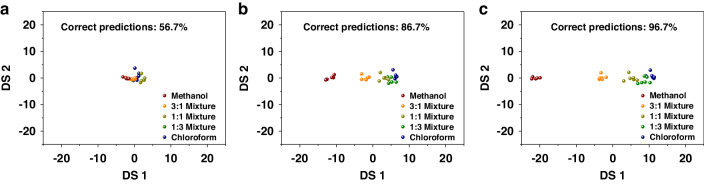
Linear Discriminant Analysis (LDA) of Binary Mixtures at Various Ratios. Discriminant plots for dual mixtures of methanol and chloroform classification using the datasets of (**a**) Δf (**b**) Δf and k_d_, and (**c**) Δf, k_a_ and k_d_

As mentioned above, the β-CD-12 functionalized sensor has the strongest discrimination capacity. Therefore, here we demonstrate the classification of dual mixtures of VOCs (methanol and chloroform) with the sensor. Mixture analytes were prepared by bubbling nitrogen gas through liquid mixtures of methanol and chloroform with volume ratios of 3:1, 1:1, and 1:3. Linear discriminant analysis (LDA) was performed for each analyte. LDA is a typical classification method, which maximizes separation between classes and minimizes objects in one class compared with PCA^55^. The discriminant results were obtained by using Δf at four input powers of the β-CD-12 functionalized sensor exposed to vapors from P/P_0_ = 0.1 to 0.6. Figure 8a shows the plot of discriminant scores (DSs) for five analytes. However, the clusters are mainly overlapped with correct predictions of 56.7%. We assume that the low discriminant validity is due to the similar response tendency for all analytes at different input powers (higher Δf at lower input power and vice versa).

**Fig. 9:**
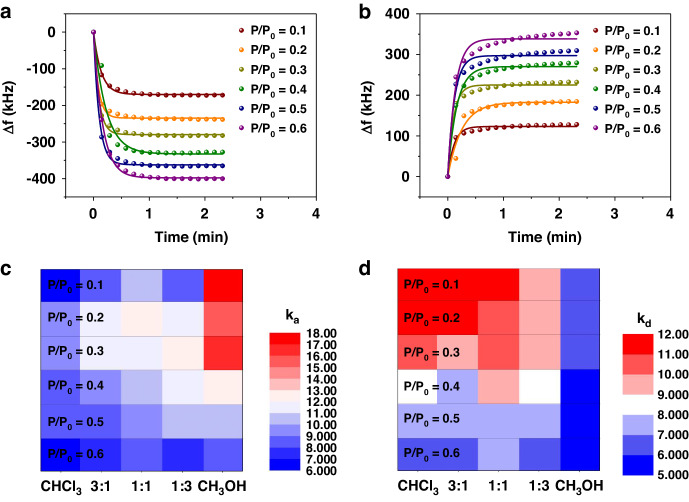
Analysis of Adsorption and Resolution Processes in Gas Mixtures. JMA fitting plots of (**a**) adsorption and (**b**) desorption process of different concentrations of 1:1 vapor mixture on β-CD-12 functionalized VSA at an input power of 10 dBm. Heat-maps of average values (**c**) k_a_ and (**d**) k_d_ for sorption processes

Multiple sensor output for VOCs detections has been used to enhance the discriminant power of VOCs sensors^27,41^. In order to further investigate the analysis performance of the sensor, two kinetics parameters, adsorption rate constant (k_a_) and desorption rate constant (k_d_), were obtained from fitting the experimental data to the Johnson-Mehl-Avrami (JMA) equation^56^.

$$\frac{\Delta {f}_{t}}{\Delta {f}_{e}}=1-\exp [-{\left({kt}\right)}^{n}]$$

where k is both k_a_ and k_d_, n is the reaction exponent and t is the reaction time. Δf_t_ and Δf_e_ represent Δf due to VOCs adsorption/desorption at t and after reaching equilibrium. As shown in Fig. 9a, b, sorption processes agree well with the JMA equation. We here use the average values k_a_ and k_d_ of sorption rate constants at four input powers as another two sensor outputs for mixture analysis. Figure 9c, d represent heat-maps which summarize average k_a_ and k_d_ for the sorption of five analytes on β-CD-12 functionalized VSA. In most cases, both k_a_ and k_d_ decrease with the increase of concentration, which indicates that it is harder to reach equilibrium with more vapor molecules in the environment. Moreover, results show that analyte with more proportion of methanol has higher k_a_ and lower k_d_ than analyte with more proportion of chloroform. It is likely that the interaction of β-CD surface with methanol molecules is stronger than that with chloroform molecules due to the similarity in hydroxy structure.

Subsequently, analytes classification was performed based on multiparameter (Δf, k_a_ and k_d_) recognition. Fig. 8b and c show the plot of DSs for five analytes using two datasets: (1) Δf and k_d_, and (2) Δf, k_a_ and k_d_. Obviously, clusters are better separated with more sensor outputs. And finally, a high discriminant validity of 96.7% correct predictions is achieved based on the second dataset. We believe that multiple parameter sensing mode provides the VSA sensor with the potential to become an e-nose system for the analysis of dual mixtures of VOCs in the future.

## Conclusion

In summary, a novel strategy for investigating the host-guest recognition process at dynamic oscillation amplitude using BAW resonators has been introduced. The molecular-scale oscillation of the resonator is beneficial for analyzing the sorptive interactions between VOC molecules and supramolecules. Four resonators modified by β-CD SAMs were used to analyze the gas-surface interactions regarding oscillation amplitude and SAM length. It can be demonstrated that higher oscillation of the resonator facilitates the desorption of vapor molecules, and molecules tend to desorb from outside of the β-CD cavity rather than from inside of the cavity during the oscillation of resonator. The differentiation of responses at dynamic oscillation amplitude enables the possibility of using a single resonator as a VSA type e-nose. Datasets of frequency responses were analyzed based on PCA and LDA algorithms for individual VOC identification and dual mixtures classification, respectively. In the case of PCA, clear clusters of different VOCs can be simply identified with longer SAM-functionalized VSA as it enhances the differentiation of responses at dynamic oscillation amplitude. In the case of LDA, mixture classification accuracies of 56.7% and 96.7% were obtained from the VSA using a single parameter (Δf) and multiparameter (Δf, k_a_ and k_d_). The VSA can process VOCs identification and classification with high accuracy by simple preparation without using a complex modification process, which is a potential candidate as a portable e-nose system for VOCs analysis in the further.

### Supplementary information

Supporting Information
