# Supplementary material for: Investigation of sorptive interactions between volatile organic compounds and supramolecules at dynamic oscillation using bulk acoustic wave resonator virtual sensor arrays
Source: Microsyst Nanoeng. 2024 Jul 17;10:99. doi: 10.1038/s41378-024-00729-x (PMC11252376; doi:10.1038/s41378-024-00729-x)
Supplement: Supplementary file 1 — Supporting Information [file 41378_2024_729_MOESM1_ESM.pdf]

Supporting Information

Investigation of Sorptive Interactions between Volatile Organic Compounds and Supramolecules at Dynamic Oscillation Using Bulk Acoustic Wave Resonator Virtual Sensor Arrays

Authors

Zilun wang[1,2,3], Zeyu Zhao[2], Suhan Jin[2], Feilong Bian[1], Ye Chang[2], Xuexin Duan[2], ,Xiangdong Men[1*],Rui You[3*]

Affiliations

[1] State Key Laboratory of NBC Protection for Civilian,Beijing 102205, China.

[2] State Key Laboratory of Precision Measuring Technology & Instruments, Tianjin University, Tianjin 300072, China.

[3]School of Instrument Science and Opto-Electronics Engineering, Beijing Information Science and Technology University, Beijing 100192, China.

* Co-corresponding author:

Xiangdong Men, Email: menxd1990@163.com;

Rui You, Email: yourui@bistu.edu.cn.

1. Device fabrication

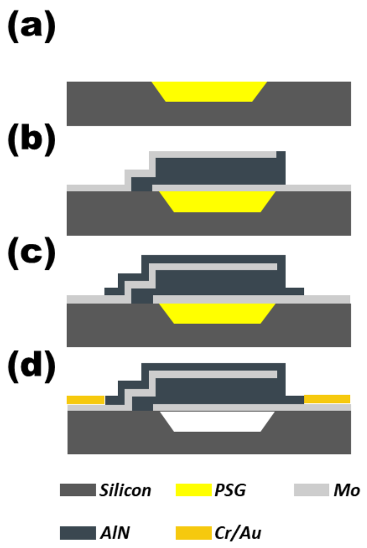

Figure S1. Schematics of the fabrication process of FBAR. (a) Etching of air cavity and deposition of passivation layer; (b) deposition of sandwiched structure; (c) deposition of passivation layer; (d) deposition of contact pads and release of PSG.

2. VOCs detection system

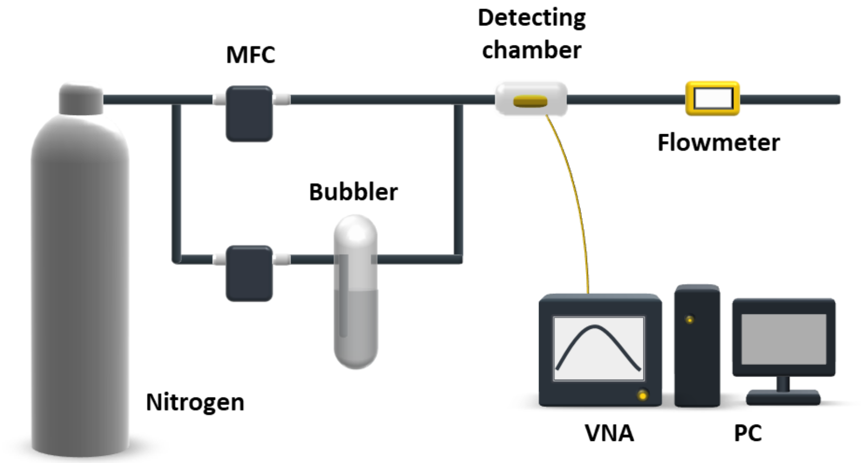

Figure S2. Illustration of the experimental setup for the detection of VOCs with FBAR VSA sensors. MFC: mass flow controller; VNA: vector network analyzer; PC: personal computer.
